# Proteomics Analysis Reveals an Important Role for the PPAR Signaling Pathway in DBDCT-Induced Hepatotoxicity Mechanisms

**DOI:** 10.3390/molecules22071113

**Published:** 2017-07-06

**Authors:** Yunlan Li, Xinxin Liu, Lin Niu, Qingshan Li

**Affiliations:** 1 School of Pharmaceutical Science, Shanxi Medical University, Taiyuan 030001, China; weiyingving@163.com (X.L.); nl88824@163.com (L.N.); 2 Department of Traditional Chinese Medicine, Shanxi University of Traditional Chinese Medicine, Taiyuan 030001, China

**Keywords:** di-*n*-butyl-di-(4-chlorobenzohydroxamato)tin(IV) (DBDCT), proteomics, PPAR signaling pathway, hepatotoxicity mechanisms

## Abstract

A patented organotin di-*n*-butyl-di-(4-chlorobenzohydroxamato)tin (DBDCT) with high a antitumor activity was designed, however, its antitumor and toxic mechanisms have not yet been clearly illustrated. Hepatic proteins of DBDCT-treated rats were identified and analyzed using LC-MS/MS with label-free quantitative technology. In total, 149 differentially expressed proteins were successfully identified. Five protein and mRNA expressions were involved in the peroxisome proliferator-activated receptor (PPAR) signaling pathway, including a scavenger receptor (CD36), adipocyte fatty acid binding protein 4 (FABP4), enoyl-CoA hydratase (EHHADH), acetyl-CoA acyltransferase 1 (ACAA1), and phosphoenolpyruvate carboxykinase (PEPCK) in DBDCT-treated Rat Liver (BRL) cells. PPAR-α and PPAR-λ were also significantly decreased at both protein and mRNA levels. Furthermore, compared with the DBDCT treatment group, a special blocking agent of PPAR-λ T0070907 was used to evaluate the relationship between PPAR-λ and its downstream genes. Our studies indicated that DBDCT may serve as a modulator of PPAR-λ, further up-regulating CD36, FABP4 and EHHADH on the PPAR signal pathway.

## 1. Introduction

**Figure 1:**
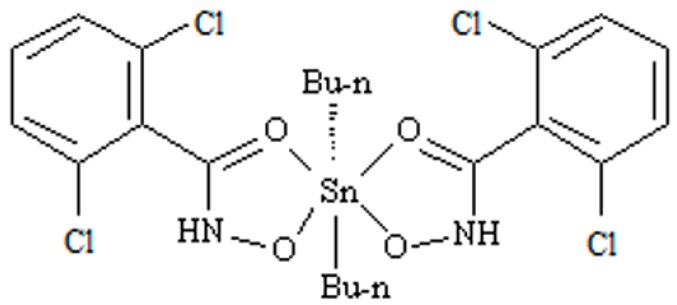
The structure of di-*n*-butyl-di-(4-chlorobenzohydroxamato)tin (DBDCT).

Proteomics has been more frequently utilized as a new method to discuss the mechanism of anticancer activity, revealing the relationship between environment and health, and diagnosing and preventing disease [1,2,3,4]. The previous study showed that the accumulation of Sn in rat livers is likely to cause hepatotoxicity. Di-*n*-butyl-di-(4-chlorobenzohydroxamato)tin (DBDCT), a novel patented diorganotin (IV) compound with a high antitumor activity, was synthesized in our laboratory [5]. DBDCT certainly observed cytotoxic activities in vitro against seven human cancer cell lines, including Hep G2, SHSY5Y, HEC-1-B, EC, T24, HeLa and A549, along with human livers, HL-7702, and a normal human hepatocytes cell; the IC_50_ values were below 10 μM when tested cancer cell lines. It also showed obvious antitumor activities in vivo against S180 and H22. Although DBDCT exhibited in vitro and in vivo antitumor activities, which, in some cases, were identical to, or even higher than, that of cisplatin, based on our former studies, it also showed notable toxicity in many rat tissues, especially in the liver tissue. The acute toxicity tests indicated that acute necrosis, focal necrosis and Kupffer cells hyperplasia appeared in the DBDCT-treated rat livers [6]. Drug-induced toxicities account for 10–52% of all causes of acute liver failures, and hepatotoxicity is the major cause of drug withdrawal and the rejection of new drug applications. It is important to further clarify the toxicology molecular mechanism for the high antitumor activity compound DBDCT. The structure of DBDCT is shown in Figure 1.

The aim of this study was to reveal differentially expressed liver proteins in DBDCT-treated rats with the help of the proteomics method. Among the technologies of proteomics, two-dimensional gel electrophoresis (2-DE) was a traditional method, which was used to identify and analyze the protein changes induced by DBDCT in our previous article [7]. However, LC-MS/MS has a higher sensitivity, better selectivity and more accuracy compared with the 2-DE method. Label-free quantitative method is considered to have higher proteome coverage capabilities [8].

Many studies have shown that hepatotoxicity is accompanied with an impaired lipid metabolism, such as a high level of triglycerides, cholesterol and fibrosis [9,10]. It was reported that tributyltin can activate the retinoid X receptor-α and peroxisome proliferator-activated receptor (RXR-α-PPAR-γ) heterodimer at nanomolar concentrations to affect the lipid metabolism [11]. In addition, CD36, FABP4, EHHADH, ACAA1 and PEPCK were the key enzymes in the process of fatty acid synthesis and transport. In this paper, it is important to notice the fatty acid metabolic process, which may have contributed to the fatty degeneration of the DBDCT-treated rat livers. Interestingly, there were seven differentially expressed proteins associated with the fatty acid metabolic process, including CD36, FABP4, ACAA1, EHHADH and PEPCK. These proteins are the downstream genes of PPAR, which may cause hepatotoxicity. Such research hints to us that DBDCT may regulate the liver lipid metabolism. In this study, LC-MS/MS and label-free quantitative methods were used to separate and identify the total proteins in the rat livers after treatment with DBDCT. Seven proteins (PPAR-α, PPAR-λ and 5 differentially expressed proteins) in the PPAR signal pathway were verified by Western blot and Reverse Transcription-Polymerase Chain Reaction (RT-PCR) assays. In a word, it was indeed important to investigate if DBDCT was a hepatotoxic agent through the PPAR signaling pathway, in view of further research and development of DBDCT as a new drug.

## 2. Results

### 2.1. Histopathology

**Figure 2:**
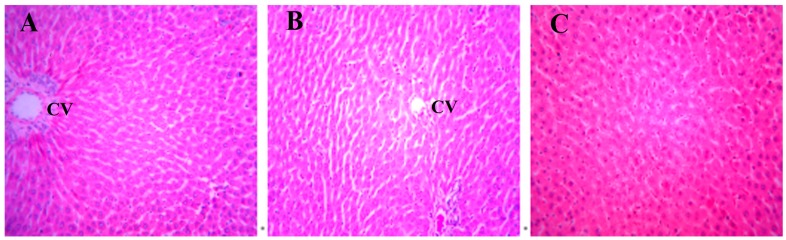
Changes in the hepatocytes stained with hematoxylin and eosin (H.E.) after DBDCT exposure (magnification: 20×). (**A**) H.E.-stained image of the control hepatocytes. A H.E.-positive reaction is evenly seen from the central vein (CV); (**B**) H.E.-stained image of hepatocytes with the treatment of the solvent. The images of the hepatocytes treated with the solvent are almost identical to those of the control group; (**C**) The images of the hepatocytes treated with DBDCT (5.0 mg/kg). After exposure to DBDCT, cell areas were swollen, and vacuoles, pyknosis, cell fusion and denucleation were visible.

A histopathology examination of rat livers was performed to verify the hepatotoxicity of DBDCT. In the control and the solvent-treated rats, the morphology of the liver tissue was normal (Figure 2A,B). Focal fatty degeneration of hepatocyte was observed in the DBDCT-treated (5.0 mg/kg) rat livers (Figure 2C). This brought up a hint that the fatty acid metabolism was abnormal in the rat livers after being treated with DBDCT.

### 2.2. Differentially Expressed Proteins in Rat Livers Exposed to DBDCT

Over 2000 proteins were identified in the rat livers exposed to DBDCT by using the effective chromatographic gradient over 85 min. Among them, about 100 were 2 times up-regulated proteins, and 49 resulted in 2 times down-regulated proteins after treatment with DBDCT (5.0 mg/kg). Table 1 shows the major biological processes with the participation of differentially expressed proteins. The results suggested that a large amount of proteins for metabolic processes displayed significant differences in expression levels in the DBDCT-treated rats, compared with the control group. It was important to notice the fatty acid metabolic process, which may have contributed to the fatty degeneration of the DBDCT-treated rat livers.

### 2.3. Pathway Data

Table 2 shows the most significant canonical pathways enriched by proteins that were significantly differentially expressed in the DBDCT-treated rats. Interestingly, there were seven differentially expression proteins that were associated with the fatty acid metabolic process, including CD36, FABP4, ACAA1, EHHADH and PEPCK. The data, including the UniProt accession ID, protein name and fold change of these proteins, are presented in Table 3. Above all, these proteins were the downstream genes of PPAR.

### 2.4. Cytotoxicities of DBDCT

**Figure 3:**
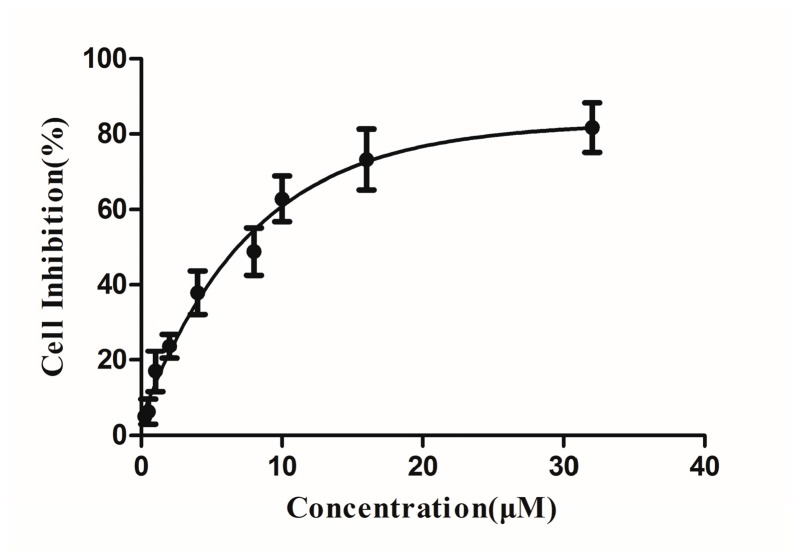
Cytotoxicities of di-*n*-butyl-di-(4-chlorobenzohydroxamato)tin (DBDCT) to Rat Liver (BRL) cells for 24 h. Results are shown as the inhibition rate, which was expressed as the mean ± SD of three experiments.

The 3-(4,5-dimethyl-2-thiazolyl)-2,5-diphenyl-2*H*-tetrazolium bromide (MTT) method was used to assess the cytotoxicities of DBDCT to BRL cells. Figure 3 shows the result of curve fitting between the cell inhibition and concentration. The IC_50_ value was 6.75 μM at 24 h, calculated by the Logit method in the GraphPad Prism software. The concentrations of DBDCT treated to BRL cells in the following experiments were projected on the basis of this data.

### 2.5. DBDCT-Induced Apoptosis Mediated by Caspase Activation

**Figure 4:**
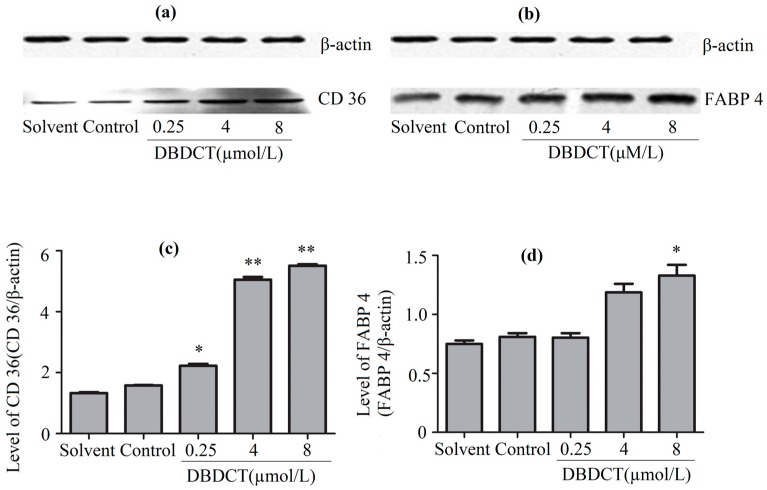
The CD36 and FABP4 expression levels of proteins in BRL cells with the treatment of DBDCT (0.25, 4, and 8 µmol/L). (**a**,**b**) The Western blot results; (**c**,**d**) Relative protein expression level of CD36 and FABP4 in cells. The results are expressed as a ratio of the expression levels of the reference protein β-actin (* *p* < 0.05; ** *p* < 0.01; compared with control group).

The CD36 and FABP4 expression levels of proteins in BRL cells, determined by the Western blot assay, are shown in Figure 4. It was demonstrated that the expressions of CD36 and FABP4 in DBDCT-treated BRL cells were both respectively increased in a dose-dependent manner compared with the control and solvent-treated groups. The result was in agreement with the observations from proteomics. It was highly probable that the rest of these three targets were differentially expressed in the DBDCT-treated cells on the protein level.

### 2.6. DBDCT Regulated the mRNA Levels of Downstream Targets in the PPAR Signaling Pathway

**Figure 5:**
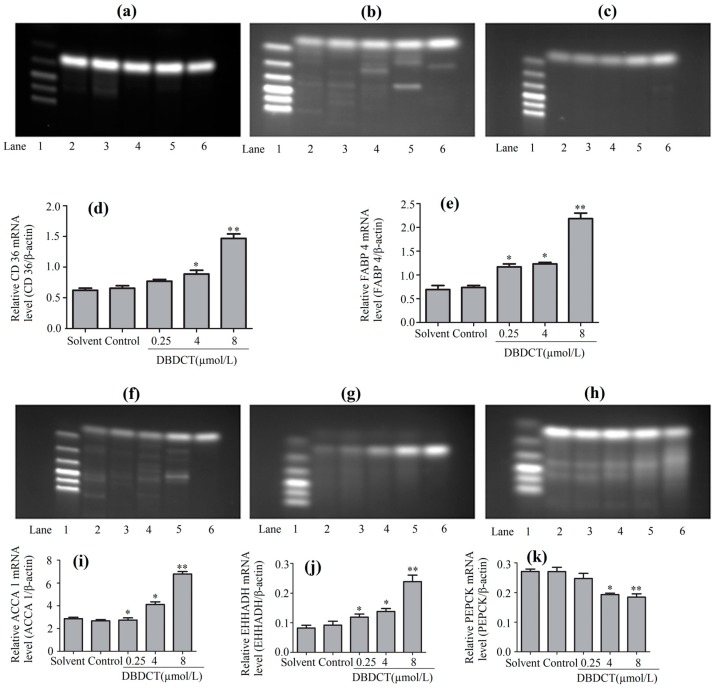
The CD36, FABP4, ACAA1, EHHADH and PEPCK expression levels of mRNA in BRL cells with the treatment of DBDCT (0.25, 4, and 8 µmol/L). (**a**–**c**,**f**–**h**) The RT-PCR results of β-actin, CD36, FABP4, ACAA1, EHHADH and PEPCK; (**d**,**e**,**i**–**k**) Relative mRNA levels of the targets in the BRL cells. Lane 1: Marker; Lane 2: Solvent; Lane 3: Control; Lane 4: 0.25 μmol/L for 24 h; Lane 5: 4 μmol/L for 24 h; and Lane 6: 8 μmol/L for 24 h. The results are expressed as a ratio of the expression levels of the reference mRNA of β-actin (* *p* < 0.05; ** *p* < 0.01; compared with control group).

To verify the change of other downstream targets in the PPAR signaling pathway, RT-PCR was performed. The mRNA levels of these targets, CD36, FABP4, ACAA1, EHHADH and PEPCK, are displayed in Figure 5. As shown in this figure, the expressions of CD36, FABP4, ACAA1 and EHHADH all significantly increased, especially those in the cells after treatment with DBDCT at the dose of 8 µmol/L. On the contrary, the mRNA level of PEPCK, markedly decreased. These results were in agreement with the findings of the protein determination.

### 2.7. Effects of DBDCT on Expressions of PPAR-α and PPAR-λ in Protein and Gene Levels

**Figure 6:**
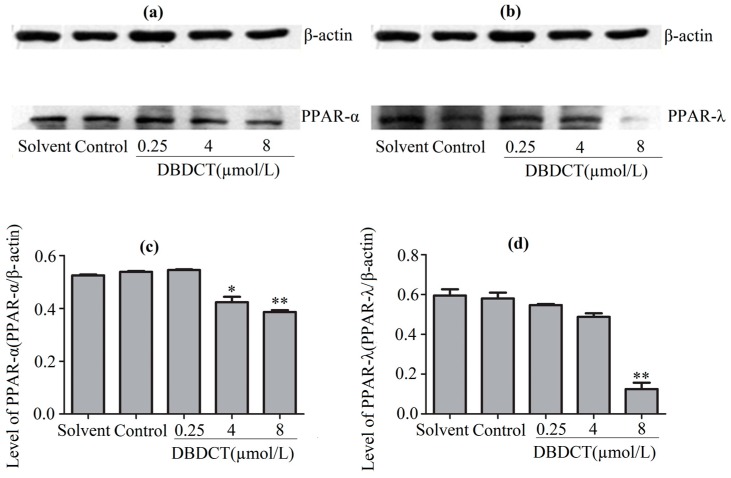
The expression levels of PPAR-α and PPAR-λ proteins in BRL cells with the treatment of DBDCT (0.25, 4 and 8 µmol/L). (**a**,**b**) The Western blot results; (**c**,**d**) Relative protein expression levels of PPAR-α and PPAR-λ in cells. The results are expressed as a ratio of the expression levels of the reference protein β-actin (* *p* < 0.05; ** *p* < 0.01; compared with control group).

**Figure 7:**
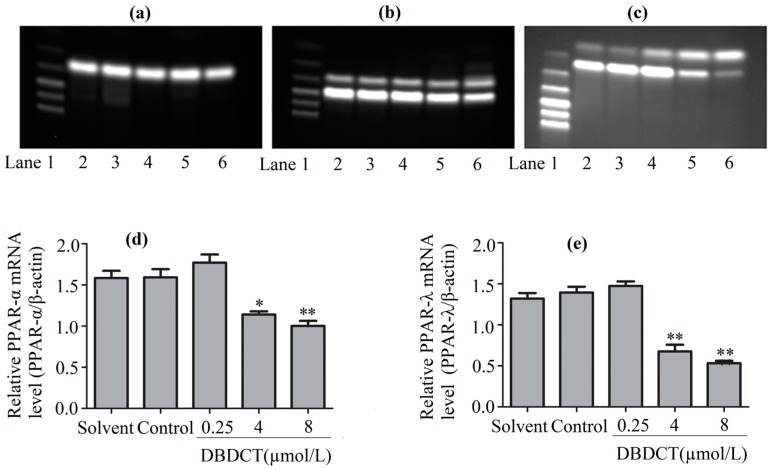
The PPAR-α and PPAR-λ expression levels of mRNA in BRL cells with the treatment of DBDCT (0.25, 4, and 8 µmol/L). (**a**–**c**) The RT-PCR results of β-actin, PPAR-α and PPAR-λ; (**d**,**e**) Relative mRNAs level of PPAR-α and PPAR-λ in BRL cells. Lane 1: Marker; Lane 2: Solvent; Lane 3: Control; Lane 4: 0.25 μmol/L for 24 h; Lane 5: 4 μmol/L for 24 h; and Lane 6: 8 μmol/L for 24 h. The results are expressed as a ratio of the expression levels of the reference mRNA of β-actin (* *p* < 0.05; ** *p* < 0.01; compared with control group).

PPARs, the regulators of the above-mentioned genes, were considered to be affected in DBDCT-treated cells on the basis of the change of these genes. We evaluated the effects of DBDCT on the expression of PPAR-α and PPAR-λ using a Western blot analysis. The results showed that the expressions of PPAR-α and PPAR-λ had obviously decreased at the doses of 4 and 8 µmol/L, compared with the control and solvent-treated groups (Figure 6). To determine whether the PPAR-α and PPAR-λ mRNA levels in cells had decreased with DBDCT as the proteins, RT-PCR was performed. It can be seen in Figure 7 that the PPAR-α mRNA levels decreased by 67.5% (*p *< 0.05) and 60.9% (*p* < 0.01), while the PPAR-λ expression decreased by 50.4% (*p* < 0.001) and 42.1% (*p* < 0.05), respectively. In a word, the expressions of PPAR-α and PPAR-λ revealed a dose-manner on both the gene and protein levels.

### 2.8. Effects of DBDCT on Expressions of PPAR-λ, CD36, FABP4 and EHHADH in Gene Levels

**Figure 8:**
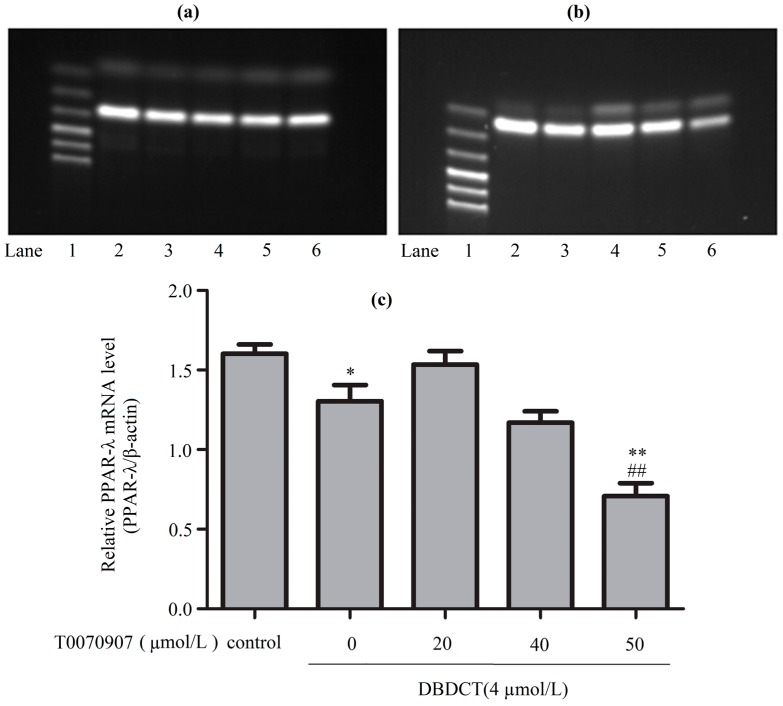
The PPAR-λ expression levels of mRNA in BRL cells with the treatment of T0070907 and DBDCT. (**a**,**b**) The RT-PCR results of β-actin and PPAR-λ (Lanes 1–6: Marker, Control, 4 µmol/L of DBDCT, 20 µmol/L of T0070907 + 4 µmol/L of DBDCT, 40 µmol/L of T0070907 + 4 µmol/L of DBDCT, and 50 µmol/L of T0070907 + 4 µmol/L of DBDCT); (**c**) Relative mRNA levels of PPAR-λ in BRL cells. The results are expressed as a ratio of the expression levels of the reference mRNA of β-actin (* *p* < 0.05; ** *p* < 0.01; compared with control group. ^##^ *p* < 0.01; compared with DBDCT group).

**Figure 9:**
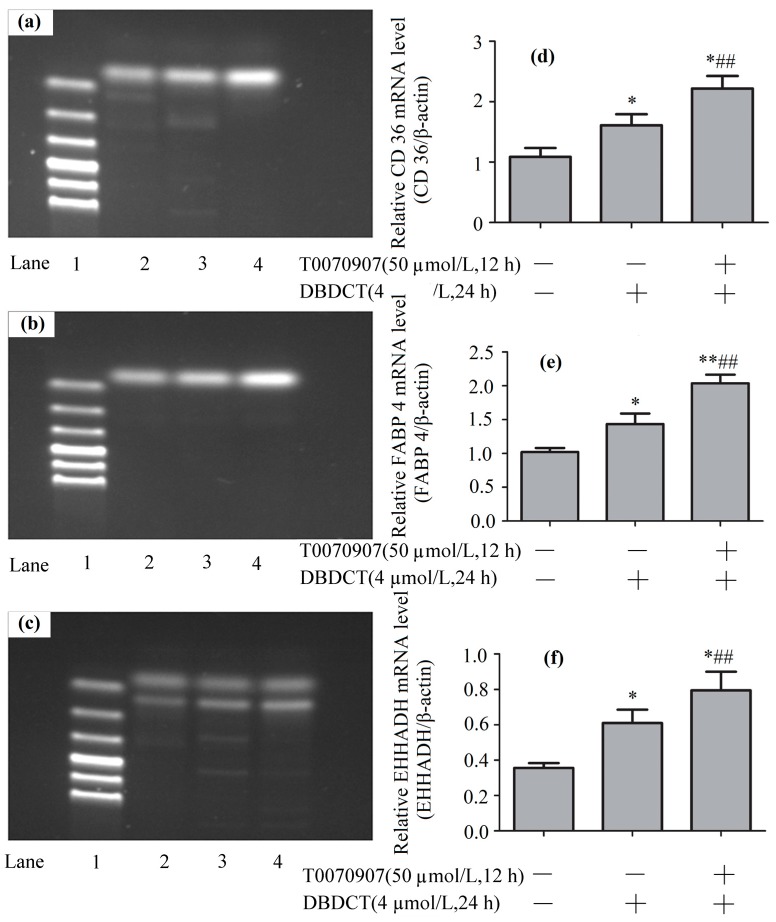
The CD36, FABP4 and EHHADH expression levels of mRNA in BRL cells with the treatment of T0070907 (50 µmol/L) and DBDCT (4 µmol/L). (**a**–**c**) The RT-PCR results of CD36, FABP4 and EHHADH (Lanes 1–4: Marker, Control, 4 µmol/L of DBDCT, and 50 µmol/L of T0070907 + 4µmol/L of DBDCT); (**d**–**f**) Relative mRNA levels of the targets in BRL cells. The results are expressed as a ratio of the expression levels of the reference mRNA of β-actin (* *p* < 0.05; ** *p* < 0.01; compared with control group; ^##^ *p* < 0.01; compared with control group).

To explore the mechanism underlying how DBDCT increased CD36, FABP4 and EHHADH by down-regulating PPAR-λ, we used T0070907 to reduce PPAR-λ in DBDCT-treated (4 µmol/L) BRL cells. Firstly, RT-PCR results (Figure 8 and Figure 9) showed that the relative PPAR-λ mRNA remarkably decreased by 54.1% (*p* < 0.05) compared with DBDCT-treated groups, where the concentration of T0070907 was 50 µmol/L. Secondly, CD36, FABP4 and EHHADH mRNA of three groups (control, 4 µmol/L of DBDCT, and 50 µmol/L of T0070907 with 4 µmol/L of DBDCT) were determined by RT-PCR. After treating with T0070907, CD36, FABP4 and EHHADH observably increased (*p* < 0.01).

## 3. Discussion

A large amount of literature has reported that the exposure of organotin to organisms altered the hepatosomatic index and hepatic triglycerides, and modulated the transcription of key lipid-regulating factors and enzymes involved in adipogenesis, lipogenesis, glucocorticoid metabolism, growth and development in the liver, revealing that organotin compounds interfere with triglyceride accumulation and the transcriptional regulation of the lipid metabolism through the activation of the peroxisome proliferator-activated receptor gamma PPARγ [12]. DBDCT, as a typical organotin compound, may also be the key process for hepatotoxicity through the activation of the PPAR signaling pathway. Recent animal research showed that the levels of alkaline phosphatase (AKP), glutamic-oxalacetic transaminase (AST) and acyl carrier protein (ACP) increased observably in DBDCT-treated rats, while the alanine aminotransferase (ALT) level decreased [6]. As the study showed, the antitumor activity of DBDCT was verified. In this study, a histological evaluation was performed to confirm steatosis of the hepatic cells in the rats injected with DBDCT by the cauda vein. The results revealed that a fatty acid metabolic disturbance was obvious in the DBDCT-treated rats’ livers. To expound adipose metabolic disorders, we performed LC-MS/MS and label-free quantitative method to analyze the total proteins of the control and DBDCT-treated rats, in order to look for the changing proteins involved in the fat metabolism; about 148 proteins were regulated. CD36 and FABP4 were determined to have increased by the Western blot analysis. This result verified the credibility of the data of proteomics. The results of biological process findings showed that there were 11 proteins in the fatty acid metabolic process. Kyoto Encyclopedia of Genes and Genomes (KEGG) analysis revealed that the PPAR signal pathway’s relevance to the fat metabolism significantly changed (*p* = 1.5 × 10^−4^). Seven differentially expressed proteins were discovered in the PPAR signaling pathway and then verified by RT-PCR. It is reported that the expressions on the mRNA level of CD36 and FABP4 were regulated by PPARs [13]. Therefore, we wondered whether DBDCT induced the change of the PPARs. The following experiment was performed to determine the expression of PPARs by a Western blot analysis and RT-PCR. The expressions of PPAR-α and PPAR-λ obviously decreased in DBDCT-treated cells. Moreover, we confirmed that there was a collaborative relationship between PPAR-λ and its downstream genes, CD36, FABP4 and EHHADH. These results initially implied that DBDCT could up-regulate some downstream genes through inhibiting PPAR-λ.

**Figure 10:**
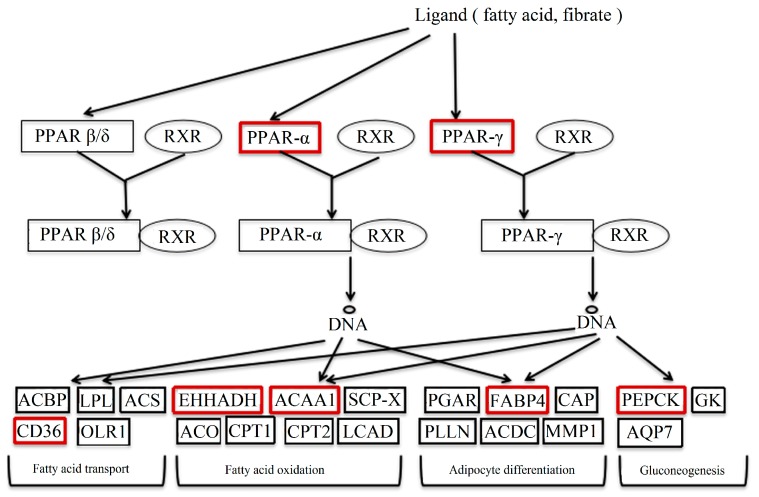
Part of the PPAR signal pathway.

PPAR is one factor of the nuclear hormone receptor superfamily, all which are induced by ligands, including unsaturated fatty acids and prostaglandins [14]. The forming heterodimer of PPAR-γ/RXR unites with the PPRE (peroxisome proliferator response element) to regulate the transcription of downstream target genes. PPARs are known to control the expression of many genes contributing to the oxidation and transport of fatty acids [15]. The partial pathway of PPAR is shown in Figure 10.

In the study, the genes in red boxes were detected. The results of these genes were verified, except for FABP5 and FABP7 because of their lesser expressions in the liver. Among these genes, CD36, FABP4, ACAA1 and EHHADH were up-regulated in DBDCT-treated BRL cells. They played important roles in the process of fatty acid synthesis and transport. CD36 regulated the ingestion and esterification of fatty acids as the receptor of fatty acids [16]. Moreover, it was reported that CD36 led to foam cell formation, which was associated with atherosclerotic lesions [17]. FABP4 participates in numbers of biological processes, such as adipocyte differentiation, lipid metabolism, the regulation of glucose, and insulin sensitivity. A recent study indicated that FABP4 induced lipid deposition in Altay sheep [18]. ACAA1, existing widely in humans and animals, catalyzed free cholesterol and long-chain fatty acids to synthesize esterified cholesterol [19]. At the same time, the expression of PEPCK on the mRNA level was markedly reduced in the DBDCT-treated rats. PEPCK has been considered as a key kinase in the biochemical regulation of glyceroneogenesis, one of the lipid metabolism processes [20]. In a word, it is inevitable to cause the accumulation of fat because of the up-regulated lipid synthesis and down-regulated lipid metabolism.

On the other hand, the decrease of PPARs, the upstream target of these genes, may have a relationship with hepatotoxicity. Firstly, PPARs play a regulatory role in the lipid metabolism. The barrier of the lipid metabolism is the key mechanism of hepatotoxicity. In this study, the downstream genes in the PPAR signal pathway were regulated by PPARs in the DBDCT-treated rats. PPAR regulates CD36, FABP4, ACAA1, EHHADH and PEPCK. For example, Bo Ma showed the reproductive toxicity and testis injuries observed due to the down-regulation of PPAR-α in testicular tissue, which subsequently led to an insufficient energy metabolism [21]. Secondly, PPREs have been proved to regulate some antioxidant genes, such as catalase (CAT) and Cu^2+^/Zn^2+^-superoxide dismutase (SOD) [22]. It was more persuasive that Nakajima T. showed that PPAR alpha-null mice exhibited marked hepatomegaly, hepatic inflammation, cell toxicity, fibrosis, apoptosis, and mitochondrial swelling after being treated with ethanol, compared with wild-type mice [23]. Clearly, PPAR is regarded as an important factor in antioxidant stress. In this study, the down-regulated PPARs could not confront the oxidative stress response, which might be a crucial mechanism of hepatotoxicity. Lastly, PPAR-α can regulate inflammatory cytokines and anti-inflammatory factors, including phosphatidylinositol 3-kinase (PI3K), osteopontin (OPN), cyclooxygenase-2 (COX-2), adiponectin and heme oxygenase-1 (HO-1) to reduce inflammation reactions [24]. Thus, the down-expression PPARs may not control inflammation reactions, which cause hepatotoxicity. Further connections of these genes to the PPAR signal pathway should be studied in the future.

## 4. Materials and Methods

### 4.1. Reagents

DBDCT was synthesized in accordance with the method reported [5], with a purity of over 99% by HPLC analysis. The stock solution of DBDCT was prepared in a solvent (90% propylene glycol, 1% ethylene diamine, and 9% saline solution), stored at 4 °C, and freshly diluted to the desired concentrations before use. Hematoxylin and eosin (HE) were purchased from Nanjing Jiancheng Bioengineering Institute (Nanjing, China). Rat BRL lines were obtained from the Cell Bank of the Chinese Academy of Sciences (Shanghai, China). Pyrolysis liquid, Dulbecco’s modified Eagle’s medium (DMEM), fetal bovine serum, the bicinchoninic acid (BCA) protein quantification, and the enhanced chemiluminescence (ECL) kits were obtained from Boster (Wuhan, China). MTT, ammonium persulfate, 30% acrylamide, tris(hydroxymethyl)aminomethane (tris), sodium dodecyl sulfate, sodium salt (SDS), tetramethylethylenediamine (TEMED), and glycine were purchased from Beijing Solarbio Science & Technology Co. (Beijing, China). Antibodies against β-actin, CD36, FABP4, PPAR-α and PPAR-λ were purchased from Bioworld Technology (Nanjing, China). The primers for β-actin, CD36, FABP4, EHHADH, ACAA1, PEPCK, PPAR-α and PPAR-λ were obtained from Shanghai Sangon Biological Engineering Technology and Service Co., Ltd. (Shanghai, China). Trizol reagent, the reverse transcript synthesis kit and 2× Taq PCR Master Mix kits were purchased from Tiangen Biotech Co., Ltd. (Beijing, China). Dimethyl sulfoxide (DMSO), formic acid, and acetonitrile were obtained from Tianjin Chemical Reagent Company (Tianjin, China). T0070907 was purchased from Selleck Chemicals (Houston, TX, USA). All other reagents used were of the purest grade available.

### 4.2. Animals’ Treatment and Tissue Collection

Wistar rats weighing 180–220 g, purchased from the Laboratory Animal Center of Shanxi Medical University, were housed in the center with conditions maintained at a temperature of 19–25 °C, a humidity of 75%, and controlled 12 h light/dark cycles. Rats in the control group were the saline group. Rats in the solvent control group were added the mixture of saline and the medium. The DBDCT-treated group was given 5.0 mg/kg once daily for 2 days, through tail intravenous injections. The drug concentration was confirmed by preliminary experiments. At the end of the 2 day experimental period, all the rats were anaesthetized. The appropriately sized livers were prepared for histological examination. The residual livers of the rats were collected and quickly frozen in liquid nitrogen for proteomics analysis. This study was carried out in strict accordance with the recommendations in the Guide for the Care and Use of Laboratory Animals of the National Institutes of Health. The protocol was approved by the Committee on the Ethics of Animal Experiments of Shanxi Medical University (Permit Number: 070101). All surgery was performed under sodium pentobarbital anesthesia, and all efforts were made to minimize suffering.

### 4.3. Histological Evaluation of Liver Tissues

The liver tissue specimens were obtained and fixed in 10% formalin for 24 h. The specimens were dehydrated, embedded by paraffin, evaluated through HE staining, and then examined using a microscope (Olympus Corporation, Tokyo, Japan).

### 4.4. Sample Fractionation

Livers of the control rats and DBDCT-treated rats were suspended in pyrolysis liquid (7 M urea, 2 M thiourea, 65 mM dithioerythritol, and 0.1% protease inhibitor) for 0.5 h on ice. The mixture was centrifuged at 12,000 rpm for 15 min at 4 °C to remove solid impurities (TD5A-WS, Changsha Xiangyi Centrifugal Machine Company, Changsha, China). Then the sample was redissolved in 0.1% formic acid. For the quantitative proteomics analysis, samples were subjected to trypsin digestion. The tryptic digest extracts were analyzed by a LC-MS/MS system according to a previous study [25]. The samples were reduced with 20 mM dithiothreitol (DTT; 56 °C, 30 min), alkylated with 50 mM iodoacetamide (26 °C, 20 min in the dark) and digested with sequencing-grade modified trypsin (1:50 *w*/*w*; Sigma-Aldrich, St. Louis, MO, USA) at 37 °C for 24 h. The solution was filtered by centrifugation at 11,000× *g* for 90 min at 4 °C in a 10 k Amicon Ultra-0.5 filter (Merck Millipore, Bedford, MA, USA).

### 4.5. Liquid Chromatography—Mass Spectrometry Analysis

Samples were analyzed on a mass spectrometer (Thermo Scientific Q Exactive Orbitrap LCMSMS, Bremen, Germany). Peptide separation from the trypsin digestion of proteins was performed using an easy-spray column (C18, 2 μm, 100 Å, 75 μm × 50 cm) at a flow rate of 250 nL/min with a linear solvent gradient to wash the column. The solvent composition was 0.1% formic acid, and 2% acetonitrile in water for channel A, and 2% acetonitrile in water for channel B. The peptides were eluted with the following gradients: 3–8% B in 10 min, 8–10% B in 55 min, 10–20% B in 30 min, 20–30% B in 15 min, 30–90% B in 20 min, and 90% B for 10 min. Mass spectrometry was accomplished in the data-dependent mode, acquiring a full-scan mass range (300–1800 *m*/*z*) after a 2.3 kV spray voltage and 250 °C capillary temperature, followed by high-energy C-trap dissociation (HCD) fragmentation. HCD spectra were acquired using an energy of 27%. Each sample was run three times.

### 4.6. Label-Free Protein Expression Data Processing

The label-free qualitative and quantitative analyses of the peptide and protein identification were performed using the Maxquant system (version 1.5, Thermo data and/or SQLite for Bruker data, Germany) Proteins were successfully identified based on 95% or higher confidence intervals of their scores’ search engines, using the following search parameters: 10 ppm for precursor ion tolerance, and 25 mmu for fragment ion tolerance; trypsin as the digestion enzyme; a fixed modification of cysteine alkylation; partial modifications of methionine oxidation and Asparagine and Glutamine taking off the amination; and two missed cleavage sites. Quantitative data were obtained with a minimum of two peptides per protein.

### 4.7. Cell Culture

Rat BRL lines were cultured in DMEM, supplemented with 10% (*v*/*v*) heat-inactivated fetal bovine serum in a humidified incubator (Heal Force HP90, Shanghai Lishen Science Technology Company, Shanghai, China), and aerated with 5% CO_2_ at 37 °C. When cells reached 70–80% confluence, they were trypsinized, counted and treated with DBDCT at the doses of 0.25, 4 and 8 µmol/L in the complete cell medium for 24 h. The cells of the solvent group were treated with the medium added with the solvent for the same duration. The cells of the control group were treated with the medium only. The two groups were both blank control groups. In addition, cells were cultured by adding different concentrations of T0070907 for 12 h (0, 20, 40 and 50 µmol/L) [26,27,28,29,30,31] and then adding 4 µmol/L of DBDCT for 24 h.

### 4.8. Cytotoxicity Assay

BRL cells were cultivated in 96-well plates. DBDCT was applied to the cells at various concentration from 0.25 to 32 μM/L for 24 h. An optical microscope (OPTECBDS200-PH, Chongqin Aote Optec Instrument Co., Ltd., Chongqing, China) was used to visualize the morphological changes of BRL cells treated with DBDCT. MTT (5 mg/mL, 10 μL) was added to each well, then cells were plated for 4 h in the humidified incubator after the medium was removed; 100 μL of DMSO was added to dissolve the resulting purple formazan after the new medium was changed, and the absorbance was read at 570 nm using an Enzyme standard instrument (Shanghai Thermo Fisher Scientifc company, Shanghai, China); a 50% cytotoxicity concentration (IC_50_) was calculated through the program of Statistical Product and Service Solutions.

### 4.9. Western Blot Analysis

The proteins were split in a cell lysis buffer, centrifuged at 4 °C for 5 min at 13,000× *g* and then kept on clear liquid. The protein concentration was determined by the BCA assay. Afterwards, the proteins (30 μg) were electrophoretically separated by 12% SDS-PAGE (6.4 mL deuterium depleted water(DDW), 5.2 mL Tris-HCl (pH 8.8), 8 mL acrylamide (30%), 0.2 mL SDS (10%), 0.2 mL APS (10%), and 0.008 mL TEMED) and then transferred to nitrocellulose membranes (Bio-Rad, Hercules, CA, USA). The membranes were first blocked with 5% non-fat milk in a Tris Buffered Saline with Tween-20 (TBST) buffer (0.2 M Tris-HCl buffer (pH 8.0), 0.1 M NaCl, 0.05% (*v*/*v*) Tween 20) for 3 h at room temperature. Then, the membranes were incubated overnight at 4 °C with rabbit polyclonal antibody anti-CD36, anti-PPAR-α or anti-PPAR-λ, at 1:1500 or 1:800 *v*/*v* dilution in the TBST buffer with 2% BSA. Following the primary antibody incubation, the nitrocellulose membrane was washed and the secondary anti-rabbit antibody (1:10,000) was suspended in the TBST buffer and placed onto the membrane for 90 min. After washing the nitrocellulose, the proteins on the membrane were detected with an ECL reagent.

### 4.10. RT-PCR Analysis

The total RNA extraction from the BRL cells was isolated by using a Trizol reagent according to the manufacturer’s instructions. One microgram of total RNA was used for reverse transcription with a reverse transcript synthesis kit. Then cDNA, the product of the transcription, was amplificated with the help of the suitable primers and the 2× Taq PCR Master Mix kits (Tiangen Biotech Co., Beijing, China). The primers and PCR conditions used for the β-actin, CD36, FABP4, ACAA1, EHHADH, PEPCK, PPAR-α and PPAR-λ amplifications were as is described below (Table 4). The PCR reaction products were analyzed by agarose electrophoresis and scanned densitometry by a gel imaging system (JY04S-3E, Guangzhou Falcon Biotechnology Co., Beijing, China).

### 4.11. Statistical Analysis

All the differential proteins were submitted to the Database for Annotation, Visualization and Integrated Discovery (DVAID) (DAVID online software: http://david.abcc.ncifcrf.gov/) to obtain the GO annotations. The pathway analysis was performed as previously described using the Kyoto Encyclopedia of Genes and Genomes (KEGG) pathway (KEGG protein database: http://www.kegg.jp/kegg/pathway.html). Data were presented as means ± SD. All experimental results were compared by the Student’s *t*-test using the GraphPad Prism program (Prism 6 for Windows, GraphPad Software Inc., San Diego, CA, USA). A value of *p* < 0.05 was considered statistically significant. The experiments were conducted with at least three replications.

## 5. Conclusions

In summary, this study utilized a proteomic approach, which combined LC-MS/MS and label-free quantitative technology, as a means to identify the differentially expressed proteins in the DBDCT-treated rats. Five proteins, CD36, FABP4, EHHADH, ACAA1 and PEPCK, were validated via Western blot and RT-PCR analyses. Because of their significantly upstream change folds, the up-regulated PPARs were determined to be reduced on the protein and mRNA level. Therefore, the PPAR signaling pathway may play a key role in the metabolism of fatty acids associated with hepatotoxicity of DBDCT. However, further studies are necessary to make sure of how PPARs are affected by DBDCT.

## Figures and Tables

**Table 1:** The major biological processes by analyzing the differentially expressed proteins.

Term	Count	*p*-Value
Oxidation reduction	31	1.2 × 10^−13^
Response to organic substance	21	6.9 × 10^−4^
Fatty acid metabolic process	11	1.6 × 10^−5^
Lipid biosynthetic process	10	1.8 × 10^−3^
Macromolecule catabolic process	10	3.8 × 10^−2^
Response to hormone stimulus	10	6.4 × 10^−2^
Protein transport	10	9.2 × 10^−2^
Establishment of protein localization	10	9.6 × 10^−2^
Response to drug	9	1.5 × 10^−2^
Cofactor metabolic process	8	3.8 × 10^−3^
Response to inorganic substance	8	1.7 × 10^−2^
Protein catabolic process	8	4.6 × 10^−2^
Lipid catabolic process	7	2.1 × 10^−3^
Di- and tri-valent inorganic cation transport	7	3.3 × 10^−3^
Steroid metabolic process	7	6.3 × 10^−3^
Cellular protein catabolic process	7	8.9 × 10^−2^
Organic acid biosynthetic process	6	1.6 × 10^−2^
Carboxylic acid biosynthetic process	6	1.6 × 10^−2^
Response to metal ion	6	3.1 × 10^−2^
Response to organic cyclic substance	6	5.7 × 10^−2^

**Table 2:** The most significant canonical pathways by analyzing the differentially expressed proteins.

Pathway	Count	*p*-Value
Drug metabolism	13	2.8 × 10^−11^
Metabolism of xenobiotics by cytochrome P450	11	1.4 × 10^−9^
Retinol metabolism	8	5.4 × 10^−6^
Linoleic acid metabolism	6	3.9 × 10^−5^
PPAR signaling pathway	7	1.8 × 10^−4^
Arachidonic acid metabolism	6	1.4 × 10^−3^
Steroid biosynthesis	4	1.7 × 10^−2^
β-Alanine metabolism	3	2.8 × 10^−2^
Pyruvate metabolism	3	7.9 × 10^−2^
Pyrimidine metabolism	4	9.7 × 10^−2^

**Table 3:** Regulated proteins in the PPAR signaling pathway identified by using the UniProt database.

Protein ID	Protein Name	Fold Change	Protein Description
Q07969	Platelet glycoprotein 4/CD36	3.56	Binds long-chain fatty acids and may function in transport and/or as a regulator of fatty acid transport. As a coreceptor fortoll-like receptor 4-6 (TLR4-TLR6), promotes inflammation in monocytes/macrophages. Upon ligand binding, such as oxLDL or amyloid-β 42 binding, rapidly induces the formation of a heterodimer of TLR4 and TLR6, which is internalized and triggers inflammatory signals, leading to the NF-kappa-B-dependent production of CXCL1, CXCL2 and CCL9 cytokines via the MYD88 signaling pathway, and CCL5 cytokine via the TICAM1 signaling pathway, as well as IL1B secretion.
P70623	Fatty acid binding protein, adipocyte 4	2.02	Lipid transport protein in adipocytes. Binds both long-chain fatty acids and retinoic acid. Delivers long-chain fatty acids and retinoic acid to their cognate receptors in the nucleus.
P07896	Peroxisomal bifunctional enzyme/enoyl-CoA hydratase	3.16	
P07871	3-ketoacyl-CoA thiolase B, peroxisomal/acetyl-CoA acyltransferase 1	2.28	
P07379	Phosphoenolpyruvate carboxykinase, cytosolic	2.02	Catalyzes the conversion of oxaloacetate (OAA) to phosphoenolpyruvate (PEP), the rate-limiting step in the metabolic pathway that produces glucose from lactate and other precursors derived from the citric acid cycle.

**Table 4:** Sequences of primers used for RT-PCR analysis and PCR conditions.

Isoforms	Primer Sequence	Product Size (bp)	PCR Conditions		
			Cycle	Denaturation	Annealing
β-actin	F:CTACAATGAGCTGCGTGTGGC R:CAGGTCCAGACGCAGGATGGC	270	30	94 °C, 30 s	55 °C, 30 s
PPAR-α	F:ACGATGCTGTCCTCCTTGATG R:GCGTCTGACTCGGTCTTCTTG	407	30	94 °C, 30 s	52 °C, 30 s
PPAR-λ	F:CCCTTTACCACGGTTGATTTCTC R:GCAGGCTCTACTTTGATCGCACD	143	40	94 °C, 30 s	59 °C, 30 s
CD36	F:TGAATCCTAACGAAGATGAGCA R:GGCTTGACCAGTATGTTGACC	106	40	94 °C, 30 s	60 °C, 30 s
FABP4	F:AGAAGTGGGAGTTGGCTTCG R:ACTCTCTGACCGGATGACGA	103	40	94 °C, 30 s	60 °C, 30 s
ACAA1	F:TGGCATCAGAAATGGGTCTT R:AGGAATCAGGCAGTCTCTGG	136	40	94 °C, 30 s	60 °C, 30 s
EHHADH	F:CACGGTTATGAGCTTGTCCA R:TCTGGCTTGCTACCTTCCTC	138	40	94 °C, 30 s	60 °C, 30 s
PEPCK	F:CTGCCTCTCTCCACACCATT R:GCCTTCCACAAACTTCCTCA	125	40	94 °C, 30 s	60 °C, 30 s

## Supplementary Materials

The following are available, Table S1: The gradient elution of UPLC by an ODS column, Table S2: Mass spectrometer parameter, Table S3: Mass spectrometer parameter.
