# Supplementary material for: Proteomics Analysis Reveals an Important Role for the PPAR Signaling Pathway in DBDCT-Induced Hepatotoxicity Mechanisms
Source: Molecules. 2017 Jul 6;22(7):1113. doi: 10.3390/molecules22071113 (PMC6152083; doi:10.3390/molecules22071113)
Supplement: Supplementary file 1 [file molecules-22-01113-s001.pdf]

molecules

Supplementary Materials:

Proteomics Analysis Reveals an Important Role for the PPAR Signaling Pathway in DBDCT-Induced Hepatotoxicity Mechanisms

Table S1. The gradient elution of UPLC by an ODS column.

Time (min)	0–10	10–65	65–95	95–110	110–130	130–140	140–150
Channel A	97–92%	92–90%	90–80%	80–70%	70–10%	10	STOP
Channel B	3–8%	8–10%	10–20%	20–30%	30–90%	90	

Table S2. Mass spectrometer parameter.

Name of Parameter	Parameter Values
Spray voltage (V)	2300
Capillary temperature (°C)	250
Collision energy	27%HCD
Full-scan mass range	m/z 300–1800
Daughter ions scan mass range	stat from m/z 100
First-level scan resolution	70000
Second-level scan resolution	17500

Table S3. Mass spectrometer parameter.

Name of Parameter	Parameter Values
Precursor ion tolerance (ppm)	10
Fragment ion tolerance (mmu)	25
Fixed modification	cysteine alkylation
Partial modifications	methionine oxidation and asparagine and glutamine taking off the amination
Missed cleavage sites	2
Digestion enzyme	trypsin
